# Assessment of Kitchen Air Pollution: Health Implications for the Residents of Ilorin South, Nigeria

**DOI:** 10.1155/2022/7689141

**Published:** 2022-08-17

**Authors:** Modinah Abdul Raheem, Ganiyat Jimoh, Halimat Abdulrahim

**Affiliations:** ^1^Department of Chemistry, Faculty of Physical Sciences, University of Ilorin, P.M.B, 1515, Ilorin, Nigeria; ^2^Department of Medical Biochemistry, Faculty of Basic Medical Sciences, College of Health Sciences, University of Ilorin, P.M.B, 1515, Ilorin, Nigeria

## Abstract

Indoor air quality is essential, so its quality cannot be compromised. Hence, this research assessed indoor gaseous air pollutant concentrations from sources in thirty-three residential kitchens within the 4-zone of Ilorin-South Local Government, Kwara, Nigeria. The work focused on SO_2_, NO_2_, and CO emission concentration quantification, determination of the air quality index (AQI), estimation of health assessment risk, and deduced their health implications on the residents. The concentrations of NO_2_ and SO_2_ were determined by the Saltzman method using a Gilair-3 air sampler, while the concentration of CO was determined using an MSA Altair-5x multigas detector. Three types of eleven kitchen environments each (kitchens where liquefied petroleum gas (LPG), charcoal, and firewood were used as fuel sources) were considered. The concentrations of NO_2_, SO_2_, and CO were higher in kitchens that used charcoal and firewood. The major health risks were deduced in percentages from the questionnaire administered, where headaches had the highest percentage (20.7). The model indicated that the concentrations of the pollutants in the evening, irrespective of the sampling points, were higher than those in the morning. Firewood contributed significantly more than charcoal and LPG (*p* < 0.05). The results of the health assessment risk showed that the risk estimated for normal exposure to the pollutants in all the households studied revealed a hazard quotient of <1.0 except for SO_2_ from firewood for infants and children = 1.09. The AQI results showed the worst health conditions for households that used firewood (0.103–4.760 ppm NO_2_; 0.327–0.647 ppm SO_2_; and 12.30–57.83 ppm CO). The study concluded that the use of LPG should be preferred as a source of fuel for cooking.

## 1. Introduction

Air pollutants, especially indoor air pollutants, are frequently regarded as a major concern of environmental health delinquents. These pollutants are continuously released from countless sources into the indoor environment. Lately, people are more concerned about the problem of indoor air quality because people spend most of their time indoors [1]. Indoor air pollution is a serious health problem. Research indicated that the concentrations of air pollutants are higher in indoor environments than in outdoor environments [2].

Indoor air pollution is a major problem in low- and middle-income nations and is reported to be responsible for 80% of air pollution exposure [3, 4]. For cooking, heating, and lighting, a variety of household energy sources are utilized around the world, including electricity, liquefied petroleum gas (LPG), kerosene, charcoal, and firewood. However, emissions from inefficient cooking fuel combustion in stoves and lights [5, 6] produce domestic air pollution (DAP), which is a significant global environmental health risk [7, 8].

In Nigeria, a lack of electricity forces people to rely on alternative power generators and kerosene for illumination, while wood and waste burning for industrial and home purposes account for more than 80% of energy consumption [9]. The high reliance on solid fuels for domestic energy among sub-Saharan African countries increases their population's exposure to the hazardous effects of air pollution [10, 11]. Cooking releases harmful pollutants (gaseous and particulates) into the kitchen from both food materials (esters, ketones, heterocyclic compounds, PAH, alcohols, ketones, alkenes, alkanes, etc.) and combusted fuels (CO, NO_x_, SO_x_, PM, CO_2_, PAH, etc.) [12, 13]. Food supplies and ingredients, fuel and stove types, ventilation conditions, cooking styles and procedures, time, and temperature are all linked to the emission of these pollutants that are harmful to the environment and human health [14].

Everyone is exposed to air pollution because breathing air is a must for living. Air pollution, even at low levels, may have an impact on human health depending on the time of exposure. Science has clearly shown from the results of some research that air pollution leads to disease, increased hospitalizations, and even premature death [15–18]. While air pollution is a complex mixture of substances, most health effects are associated with the major components of smog, fine particulate matter (PM), ultrafine particles (UFPs), total volatile organic compounds (TVOCs), nitrogen dioxide (NO_2_), ozone (O_3_), and sulphur dioxide (SO_2_) [15, 19]. Epidemiologic studies have linked air pollutants such as O_3_, SO_2_, and NO_2_ to increased health challenges and mortality [20]. Prolonged exposure to these pollutants is implicated in the pathogenesis of neoplastic changes such as lung cancer, skin cancer, and other diseases related to loss of control in cell cycle regulation [21, 22]. A number of air pollutants such as NO_2_, SO_2_, O_3_, carbon dioxide (CO), volatile and semivolatile organic compounds (VOCs), PM, radon, and microorganisms have been recognized to exist indoors [20]. Some of these pollutants (NO_x_, SO_2_, O_3_, PM) are common in both indoor and outdoor environments, and some of them may have originated from outdoors. These air pollutants can be inorganic, organic, biological, or even radioactive. The effect of these air pollutants on humans depends on their toxicity, concentrations, and exposure time and may vary from person to person [20, 23]. Indoor air pollutants affect the aged, children, infants, and adults, with increased effects on children and the elderly. Air pollution emanates from various sources, which include natural and anthropogenic ones. Natural sources of air pollution include volcanoes, which produce sulphur, chlorine, and particulates. Wildfires result in the production of smoke, carbon dioxide (CO_2_), and carbon monoxide (CO). Most forms of air pollution are because of human activities and they include fossil fuel burning (charcoal, oil, and natural gas as a source of heating), electricity generation, vehicle emissions, aircraft emissions, domestic fuel burning, and the use of household materials that contain persistent organic pollutants, biomass burning, and waste incineration [24].

Common indoor air pollutants, which include carbon (II) oxide (CO), sulphur (IV) oxide (SO_2_), nitrogen (IV) oxide (NO_2_), volatile organic compounds (VOCs), formaldehyde (HCHO), and particulate matter (PM) from charcoal, firewood, and LPG, have ripple effects on health with low ventilation, seasonal variations, metrological factors, and other environmental factors [25, 26]. Carbon (II) oxide is a tasteless, odorless, colorless, and nonirritating gas produced by incomplete combustion of organic materials and is the leading cause of poisoning in the United States [27], which could be in any other region because of its high affinity with hemoglobin in the blood system of the residents present in indoor air with no or less cross ventilation. Sulphur (IV) oxide is a primary combustion product of fossil fuels that is usually grouped together with acid aerosols and particles to form a complex group of distinct air pollutants associated with a wide array of adverse health effects, including short-term respiratory morbidity and mortality [28]. NO_2_ is a gas that is light yellowish-orange at low concentrations and brown at high concentrations. It has a pungent, irritating odor and is extremely corrosive, especially in wet environments [29]. The pollutants enter the body by absorption through the skin and mucous membranes. Through injection and inhalation, men can exchange large amounts of gaseous pollutants everyday via the lungs, thereby affecting the respiratory system [30]. Air pollution significantly affects human health and the ecosystem [31, 32]. Research indicates that global deaths directly or indirectly attributable to ambient air pollution reached almost 4.5 million in 2015 [32], while the Global Burden of Disease study showed that about 6.7 million premature deaths were recorded due to indoor and outdoor air pollution from anthropogenic and natural sources [33]. Exposure to ambient PM2.5 is estimated to cause 4.2 million uncertainty interval (UI) 3.7, 4.8 deaths worldwide each year [32, 34]. The air quality index (AQI) is calculated for each air quality parameter according to the following modified formula [35]:

(1) $$\begin{array}{c} AQI=\frac{I_{HI}}{BP_{HI}}-\frac{I_{LO}}{BP_{LO}}X\left(C_{M}-BP_{LO}\right)+I_{LO}. \end{array}$$

  AQI = Air quality index.
  I_LO_ = Index at the lower limit of the AQI category.
 
*I*_HI_ = Index at the upper limit of the AQI category.
  BP_LO_ = Break-point concentration at the lower limit of the AQI category.
  BP_HI_ = Break-point concentration at the upper limit of the AQI category.
 
*C*_M_ = 8-hour pollutant concentration.

The equation has been used to determine the AQI values for ratings in the range of 0–500, where 0–50 is good, 51–100 is moderate, 101–150 is unhealthy for sensitive groups, 151–200 is unhealthy, 201–300 is very unhealthy, and 300–500 is hazardous. However, USEPA [36] has interpreted these AQI values for pollutants in the concentration of ppm as shown in Table 1.

Several studies on indoor residential air quality have been conducted, though not in the form reported in this study, but there is a scarcity of data on residential building indoor air pollution and its health implications in Ilorin, Kwara state, and Nigeria as a whole. Hence, this research aimed at quantifying the concentrations of NO_2,_ SO_2,_ and CO from residential kitchens within the sample population selected for this research study, assessed the air quality index, and evaluated the implications for the health of the residents. The data were modeled so that the study's findings could serve as a baseline for indoor air pollution knowledge in the Ilorin South Local Government Area, Kwara State, Nigeria, and beyond.

## 2. Experimental Design

### 2.1. Sampling Area

Ilorin South is a local government area in Kwara state, Nigeria, with Its headquarters in Fufu town. It has an area of 174 km^2^ and a population of 208,691 at the 2006 census. It consists of 11 wards (http://new.489.life/demoss/docs/wards-in-ilorin-south-ab115a) as shown in Table 2. The wards in the local government region are not situated in the same region; rather, they are interwoven. Table 2 shows the four major zones of the local government, with each zone further divided into wards. Akanbi zone (I) has five wards, Balogun Fulani zone (II) has three, Okaka zone (III) has two, and Oke Ogun zone (IV) has one. These accounted for the eleven wards within the four zones under the Ilorin South Local Government of Kwara state, Nigeria.

Within these eleven wards, three households each were sampled, each with LPG, charcoal, and firewood as sources of fuel for cooking in their kitchens, totaling thirty-three (33) household kitchen populations taken as sampling points. The pollutants emitted from the fuel sources used in cooking in these kitchens were collected for further studies and are reported in this work. The coordinates of these sample kitchens, zones, and wards where they belong within the local government of interest are as shown in Table 2.

### 2.2. Sample Collection

The World Health Organization (WHO) [37] defined indoor air quality (IAQ) as the physical and chemical nature of air, as delivered to the breathing zone of building occupants, which produces a complete state of mental, physical, and social wellbeing of the inhabitants, and not merely the absence of disease or infirmity. In commercial buildings, indoor air quality arises when there is an insufficient quantity of ventilation air being provided for the amount of air contaminants present in the conditioned space.

Indoor kitchen data were collected in a local government area in four zones (z) of eleven wards and thirty-three (33) residential buildings during two times of the day (morning and evening). The residential buildings were selected in a way that their kitchens belonged to low- (11), middle- (11), and high- (11) income earners. For example, LPG, charcoal, and firewood were sources of fuel used for cooking by low-, middle-, and high-income earners, respectively. The buildings of low- and middle-income earners have 8 to 10 rooms with different occupants (families) with detached kitchens, while those of high-income earners contain flats with fewer rooms owned by a family with attached kitchens. The afternoon period was not included because households seldom cook in the afternoon due to the nature of their jobs, irrespective of the income class they belong to. The sampling was done at an integrated 3 hours each in the morning (6 : 00–9 : 00 a.m.) and evening (6 : 00–9 : 00 p.m.) across the sampling points, in which 11 households each used LPG, charcoal, and firewood for cooking. Some parts of the buildings were located close to the road, market, and public places, while some parts were located in urban and rural areas because of the nature of the local government used as a case study. Unlike other households that serve as the population samples for this study, only two of the kitchens in the households that used LPG for cooking had poorly ventilated kitchens, and most of the residents are prone to inhalation of smoke.

### 2.3. Calibration

The MSA Altair 5x multigas sensor is embedded with a software design that meets the requirements of the International Electrotechnical Commission (IEC, 61, 508–3), which is the international standard for electrical, electronic, and programmable electronic safety-related systems, guaranteeing reliable and dependable operation and certified by Lloyds Register. It offers flexibility, assurance, and robustness [38, 39].

Cascade Controls Limited (Oilfield) performed the calibration every six months since the last one, and certificate number CCL/TC/209/19 was issued. The unit of the sensor is in ppm. In the field, immediate field calibration of the MSA Altair 5x sampler unit was performed by first switching it on in a controlled environment to perform the bump test, which zeros the sampler unit. MSA Altair 5x then displayed the sensor and calibration information, which completed the fresh air setup. The sensor described above was used for indoor sampling by measuring the pollutants for 3 hours at a point located 2 m away from the kitchen edge at each sampling point located along the residences under study.

### 2.4. Measurements of SO_2_, NO_2_, and CO

The GilAir 3 high volume sampler and the MSA Altair 5x multigas sensor were placed inside the kitchen of each sampling site to monitor indoor air pollutants (SO_2_, NO_2_, and CO). The GilAir 3 was used to monitor NO_2_ and SO_2_, while the MSA Altair 5x multigas sensor was used to monitor CO. The sampler was placed in the direction of the wind and at a height where the pollutants were easily trapped [40]. NO_2_ and SO_2_ were trapped by preprepared selective absorption solutions with GilAir 3. The sampler had an adjustable flowmeter part which was used to measure the volume of air that was drawn through the pressure difference into the absorbing solution. All samples for both pollutants were taken by bubbling air through a specific absorbing reagent, that is, a gas chemically selective absorbing solution, using the GilAir 3 air sampler, which works under the same principle as Lamotte [41]. The flow rate was maintained at a very low speed (0.33 L/m) because the smaller the bubbles, the more surface contact was permitted between the gas and absorbing solution, and a higher efficiency of gas absorption resulted [42].

The concentration of NO_x_ pollutants was inferred by calculations from the pollutants' absorbance measured at 550 nm with the aid of a double-beam UV-visible spectrophotometer (model UV-6300PC). The absorbance was later converted using the Beer–Lambert equation. The law of Beer–Lambert relays the light absorbed by a solution to the properties of the solution according to this equation: *A* = *ε*bc, where A is the amount of light absorbed by the solution at a given wavelength, the molar absorptivity of the absorbing species (*ε*), the path length (b), and the concentration of the absorbing species (c). The detection limit for NO_2_ is 10–2 ppm.

The concentrations of SO_2_ were recorded using a conductivity meter (Model HI-2030-02 Edge Bench) in mScm-1 and then converted to parts per million (ppm) with a detection limit of 0.3 ppb.

The concentrations of CO were recorded directly from the CO sensor in ppm from the MSA Altair 5x multigas sensor used. The data were taken over 3 hours, and the average was found to have a detection limit of less than 50 ppm.

### 2.5. Hazard Identification

The identification of CO, NO_2_, and SO_2_ as harmful and their associated health risks were performed through a review of existing literature [43]. Risk characterization is the quantitative estimation of the health risk of exposure to a pollutant. Here, an estimate of possible noncarcinogenic effects from exposure to a known pollutant is determined using the hazard quotient (HQ) [43]. It reflects the probability of an adverse health outcome occurring among healthy and/or sensitive individuals. Noncancer risks were calculated for acute and chronic exposure scenarios as follows:

(2) $$\begin{array}{c} HQ=\frac{ADD}{REL}\left(\text{chronic exposure}\right)\text{or}HQ=\frac{AHD}{REL}\left(\text{acute exposure}\right), \end{array}$$

where “reference exposure level” (REL) is the dose at which significant adverse health effects will occur in exposed groups compared with the unexposed group. ADD is the average daily dose of the pollutants (*µ*g/kg/day) and AHD is the average hourly dose per day.

In this study, the term “REL” was used as adopted by the Office of Environmental Health Hazard Assessment (OEHHA). An HQ of 1.0 is considered the benchmark of safety. An HQ that is < 1.0 indicates a negligible risk, that is, the pollutant under scrutiny is not likely to induce serious adverse health effects, even on a sensitive individual. An HQ > 1.0 indicates that there may be some risk to sensitive individuals as a result of exposure [44, 45].

The data obtained in this research for mean body weight were similar to the one obtained from Reference [45], which is the reason for the adoption of the data.

For exposure to noncarcinogenic pollutants (CO, NO_2_, SO_2_), the acute exposure rate equation is given as follows:

(3) $$\begin{array}{c} \text{AHD}=Cx\frac{IR}{BW}, \end{array}$$

where AHD denotes the average hourly dose for inhalation (g/kg/hour), C denotes the pollutant concentration (g/m3), IR denotes the inhalation rate (m3/hour), and BW denotes the body weight (kg) [46].

### 2.6. Questionnaire

A questionnaire that includes two sections (section A contains their personal data; gender, type of family, kitchen, level of education, income, cooking times, etc., while section B focuses on health-related questions) was developed for the assessment of information from the residents on housing characteristics, lifestyle related to indoor air pollution variability, and the health challenges of pollutants. Thirty-three (33) questionnaires were self-administered to the households and used as a case study.

### 2.7. Statistical Analysis

Results from the experiment and questionnaire were statistically evaluated using Microsoft Excel 2010 software and IBM SPSS statistics (version 21). SPSS was used to evaluate the mean and analysis of variance (ANOVA) for the set of results. ANOVA shows significant differences within a data set. All procedures were carried out in triplicate to ensure reliability, and samples were carefully handled to avoid contamination. All glassware was properly cleaned and all reagents used were of analytical grade. To avoid contamination of the procedure, double distilled water was used.

## 3. Results and Discussion

The concentrations of the samples were averaged to represent the 11 wards (Akanbi I–V, (z1); Okaka I&II, (z2); Balogun Fulani I-III, (z3); Oke Ogun, (z4)) to facilitate discussion of the results and to express pollution across the four zones and the Ilorin South Local Government Area of Kwara state, Nigeria. The concentration exposure time was done for a short term over 3 hours (less than 8 hours) [34] and compared with a 1-hour indoor standard exposure limit of 0.1 ppm NO_2_; 0.133 ppm SO_2_; and 25 ppm CO [47–49]. The longer the sampling time, the less the concentration of pollutants that are absorbed. The HQ values are compared to <1 as a negligible risk to all groups; = 1 as a safety benchmark; and >1 as a risk to sensitive groups due to exposure.

### 3.1. Concentrations of NO_2_ in Parts Per Million (PPM) and Their AQI Rating in Selected Households across the Sampling Points

The concentration of NO_2_ was measured and analyzed in 3 selected households in which firewood, charcoal, and LPG fuel were used as sources of fuel in each sampling area of the 4 zones in the Ilorin South Local Government Area of Kwara state. The average concentration of NO_2_ in ppm and their air quality index in selected households where LPG, charcoal, and firewood were used for cooking are presented in Table 3.

The air quality indices for households that used LPG and charcoal for cooking across the sampling points are good with little potential to affect public health, while it is unhealthy for sensitive people in households that used firewood. The major contribution to the indoor pollution was from firewood and charcoal, with the concentration of gaseous pollutants being low in the households that used LPG as a source of fuel for cooking. The concentrations of indoor pollutants from firewood ranged from 0.077 to 0.476 ppm and were found to be higher than the indoor air quality standard [47], with a 1-hour limit of 0.1 ppm. The NO_2_ concentration in kitchens that used firewood was higher than the concentration of NO_2_ in kitchens that used charcoal. Similarly, the concentration of charcoal is higher than the concentration emitted from LPG. These results conformed to the results reported by the researcher and his co-workers [50], who also added that the concentrations of pollutants are significantly high in indoor environments and usually exceed the air quality standard stipulated for outdoor environments. The highest average concentration was recorded from firewood usage in sampling z2 in the evening, which could be a result of the confinement of space, the closeness of the sampling points to markets and public places, as well as proximity to the main road. The lowest concentration of the pollutants was observed in the household where the source of energy for cooking was LPG (0.012–0.101 ppm). The results fall within the limit stipulated. All emitted concentrations of NO_2_ from households that use firewood and charcoal as fuel exceeded the indoor set standard of 0.1 ppm [47].

In general, the concentrations of NO_2_ from households that use LPG across the sampling zones are within the limit. The concentrations of NO_2_ from households that used charcoal across the sampling zones ranged from 0.053 to 0.122 ppm. The highest concentration (0.122) was observed in z1 at SP 4. This could be a result of the closeness of the households in this sampling area to the market, confinement of places, or proximity to the road, while the lowest concentration (0.053) was observed in z2 at SP 7, the zone that has the lowest population. The concentration of NO_2_ in households that used firewood ranged from 0.077 to 0.476 ppm. The lowest concentration (0.077 ppm) was observed in z4 at SP 11. The activities were similar to those of households that used charcoal for cooking. The highest concentration of NO_2_ was observed in households that used firewood in z2 at SP7. Apart from the population, this could be attributed to a number of factors, including the proximity of the inhabitants' homes to the road, the predominance of low-income earners, residents' daily activities, poor ventilation, and proximity to markets and public places. Despite their small population, more than 80% of the people in this area use firewood as a source of cooking fuel, which may emit a similar high percentage of air pollution into the environment. This is similar to the report of the authors of [3,4] who studied low- and middle-income residents, where firewood and charcoal were used as a source of generating heat for cooking. The HQ values of the local government used in this study are less than 1. The kitchen that used firewood had an overall score of 0.71 for infants and children, which is still low compared to the risk benchmark of 1. As reported [42], exposure to smoke is arguably the greatest indoor air pollution problem in developing countries, of which Nigeria is one. LPG as a source of fuel contributed very low concentrations to the NO_2_ emission of indoor air pollutants across the studied sampling points.

### 3.2. Average Concentration of SO_2_ in PPM and Their Air Quality Index in the Selected households

The concentration of SO_2_ was absorbed in different households that used LPG, charcoal, and firewood as sources of energy for cooking in the sampling points and was analyzed. The average concentrations of SO_2_ in ppm and their air quality index in these selected households averaged over the sampling points are shown in Table 4.

The results in Table 4 show that the AQI for households that used LPG, charcoal, and firewood for cooking across the sampling points was dangerous to their health. The SO_2_ concentrations in selected households across the sampling points are not within the 1-hour limit (0.133 ppm) set for indoor air quality minimum exposure. The highest concentration (0.647 ppm) of the pollutant was observed in the household that used firewood in the evening period in z1 at SP 5, which far exceeds the limit (0.133 ppm) set in almost 5 folds; with this, and the remaining 4 households, the residents are not safe since the pollutants can cause serious danger to their health. This observation supports the findings of [51–59] where NO_2,_ SO_2_, and CO content in air pollutants are recognized as a major cause of one or more disease-related ailments (irritation of mucous membranes, difficulty in breathing, and change in behavior). Given the level of SO_2_ emissions in this study area, measures to monitor and ensure compliance with air quality guidelines recommendations should be anticipated. The lowest concentration was recorded from a household in z1 at sampling point 2 (0.150 ppm) in the morning for the household that used gas as their source of generating heat. The low concentration could be attributed to the household being located in a rural area, the presence of ventilation, and distance to public places, although the concentration is still unhealthy for sensitive groups [50]. As indicated in the results (Table 4), the daily concentrations of SO_2_ across the sampling points range from 0.150 to 0.647 ppm, which have health implications that range from unhealthy for sensitive groups to very unhealthy to hazardous [50]. The SO_2_ concentration in households that use charcoal and firewood ranged from 0.237 to 0.491 ppm and 0.327 to 0.647 ppm, respectively.

Since the examined residents are mostly low-income earners, their constant use of firewood contributed to the increase in the pollutant concentrations, and it equally affects very few households that use gas because the emissions travel by air through advection. Firewood has the highest SO_2_ emission concentration, followed by charcoal. The HQ value of the local government used in this study is less than 1. The kitchen that used firewood had an overall rating of 1.09 for infants and children, which is above the safety benchmark of 1. Exposure to smoke is arguably the greatest indoor air pollution problem [42]. Cooking with LPG contributes to SO_2_ pollutants, although at a relatively lower concentration, which is equally unhealthy for sensitive groups.

### 3.3. Average Concentration of CO in PPM and Their Air Quality Index (AQI) in the Selected Households

The concentration of CO was also measured in selected households in which firewood, charcoal, and gas were used as sources for cooking heat in the sampling points and analyzed. From Table 5, the air quality indices for households that used gas for cooking across the sampling points are good with little potential to affect public health, while it is dangerous for residents in households that used charcoal and firewood for cooking. The CO concentrations emitted from gas fuel in the morning and evening across the sampling points are within the limits stipulated by [37] 1-hour (10 ppm) and [36] 1-hour (25 ppm).

There was an increase in the concentrations of the pollutant in the evening at all sampling points due to the increase in activities in the afternoon towards the evening period. The highest concentration of pollutants was observed in the household that used firewood in z1 at SP 1 (57.83 ppm), which also exceeded the stipulated standards set. The sampling points with charcoal and firewood have higher concentrations of the pollutants except for z1 and SP 3, with 9.90 to 13.32 ppm, which is only unhealthy for sensitive groups.

Exposure to smoke is arguably the greatest indoor air pollution problem [42]. Gas sources emitted the lowest concentration when compared to charcoal and firewood, and the concentrations of the pollutants increased in the evening due to the increase in activities. The air quality in the households across the sampling points that use charcoal and firewood is very unhealthy and hazardous, respectively. Because of this, the residents are not safe since the pollutants can cause serious danger to their health. As can be deduced from Table 5, the daily average concentration of CO across the sampling points ranged from 3.01 to 57.83 ppm. The lowest concentration (3.01 ppm) was observed in z2 at SP 7, while the highest average concentration (57.83 ppm) was observed in z1 at SP 1. The CO concentration in households that used charcoal and firewood ranged from 9.9 to 57.83 ppm. The major source of indoor air pollutants was firewood and charcoal. The high concentration of CO may alter important body functions such as oxygen exchange in the lungs, or oxygen transport in the blood and delivery at the tissue level. Irritant pollutants may lead to irritation and long-term damage to the eyes, nose, throat, and mucosal lining/surfaces of the body [60]. The average range of atmospheric concentrations of some pollutants in some households in the Lagos metropolis was found to exceed the standard [61]. Some research studies also reported that the use of charcoal and cook stoves during indoor cooking accumulates high concentrations of pollutants, particularly in the indoor environment [60–62]. With the concentration of CO values reported, it produces some negative effects among people with asthma and other respiratory issues in most households when there is repeated exposure over a long period of time, which can cause health effects like dizziness, headaches, redness of the eyes, and acute respiratory effects as noticed and captured through the questionnaire during the period of sampling. However, excessive CO concentrations may cause chronic obstructive pulmonary disease (COPD), chronic bronchitis, adverse reproductive outcomes, and pregnancy-related problems such as stillbirth, low birth weight, and lung cancer in some cases [63]. The HQ values used in this study are less than one, though the kitchen that used firewood had an overall of 0.44 for infants and children, which is still low compared to the safety benchmark of one.

### 3.4. Estimation of Parameters with Dummy Multilinear Regression (MLR)

To carry out regression analysis in fitting the model for the data acquired in this research, sampling zone 1 (z1) is thereby centered as the reference category in terms of the sampling area. Based on time, “morning” is thereby centered as the reference category, while “gas” as a cooking method is centered as the baseline category among others. The concept of assigning a value to a dummy variable is such that the categorical independent variable takes the value of 1 wherever it corresponds to the value of the dependent variable and a value of zero (0) where otherwise.

#### 3.4.1. The Full Model

OKA stands for Okaka (z3), OKGN stands for Oke-Ogun (z4), EVNG stands for evening, CHAR stands for charcoal, and FIWD stands for firewood. Y-dependent variable (pollutant concentration), *α*-coefficient of sampling point, *θ*-coefficient of time, *γ*-coefficient of method of cooking, *β*-coefficient of the sampling zones, z2-sampling zone 2, z3-sampling zone 3, z4-sampling zone 4.

#### 3.4.2. Reference Category

Sampling zone–Sampling zone 1 (z1), Time-morning, Cooking Method–Gas.

The model fit resulted in the following equation:

(4) $$\begin{array}{c} \text{Concentration}\left(y\right)=\alpha +\beta_{1}\left(BAL\right)+\beta_{2}\left(OKA\right)+\beta_{3}\left(OKGN\right)+\theta_{1}\left(EVNG\right)+\gamma_{1}\left(CHAR\right)+\gamma_{2}\left(FIW\;D\right)+e_{ij}. \end{array}$$

Table 6 shows that the concentrations of NO_2_ in z2 and z3 are higher than that of z1 by 0.029 and 0.020 ppm, respectively, while the z3 concentration of NO_2_ is less than that of z1 (reference category) by 0.049 ppm. In terms of period, the concentration of NO_2_ in the evening, irrespective of the sampling point, is 0.007 ppm higher than in the morning (reference category).

Based on the cooking method, the concentration of NO_2_ of using charcoal and firewood is higher than that of gas (reference category) by 0.039 ppm and 0.177 ppm, respectively. This result subsequently showed that the concentration of NO_2_ from burning firewood is higher than that of charcoal. According to the model, charcoal and firewood contribute more to the concentration of pollutant NO_2_ than gas cooking sources. According to the model, firewood contributes significantly more (p 0.05) to the concentration of pollutant NO_2_ than charcoal and gas cooking methods.

Based on the findings, Table 7 shows that the concentrations of SO_2_ in z2 and z3 are less than that of z1 by 0.006 ppm and 0.008 ppm, respectively, while the z3 concentration of SO_2_ is approximately equal to that of z1 (reference category).

In terms of period, the concentration of SO_2_ in the evening, irrespective of the sampling point, is 0.009 ppm higher than in the morning (reference category).

According to the cooking method, the concentration of SO_2_ from using charcoal and firewood is higher than that of gas (reference category) by 0.032 ppm and 0.059 ppm, respectively. This result also showed that the concentration of SO_2_ from using charcoal is slightly less than that of firewood. According to the model, charcoal, and firewood contribute significantly more (p 0.05) to the concentration of the pollutant SO_2_ than gas cooking.

In Table 8, the concentrations of CO in z2 and z3 are higher than those of z1 by 1.1 and 3.3 ppm, respectively, while the z3 concentration of CO is less than that of z1 (reference category) by 1.4 ppm. On the basis of time, the concentration of CO in the evening, irrespective of the sampling point, is 4.7 higher than the concentration of CO in the morning (reference category) in ppm.

Based on the cooking method, the concentration of CO as a result of using charcoal and firewood is higher than that of gas (reference category) by 21.7 ppm and 30.4 ppm, respectively. This result subsequently showed that the concentration of CO from using firewood is higher than that of charcoal. The model indicates that charcoal, as well as firewood, contribute significantly more to the concentration of pollutant CO than the gas cooking source method and that the concentration of pollutant CO is significantly higher in the evening (*p* < 0.05).

The respondents in the households under study, as shown in Table 9, were discovered to have cough (17.2%), dizziness (9.2%), irritation of the eye (11.5%), headache (20.7%), and sneezing (11.5%) as the major health risks associated with exposure to pollutants. The ailments are in agreement as reported in the literature [31]. The most common effect is called sick building syndrome (SBS), in which people experience uncomfortable or acute health effects such as irritation of the nose, eyes, and throat, skin ailments, allergies, and so on [64]. These ailments are the major symptoms of acute respiratory tract infections and asthma, which alter important body functions such as oxygen exchange in the lungs or oxygen transport in the blood. Irritant pollutants may lead to irritation and long-term damage to the eyes, nose, throat, and other wet surfaces of the body; they enter the body by absorption through the skin, ingestion, and inhalation [65]. It was discovered that poor breathing (3.4%) and cough (17.2%) primarily affected the elderly, whereas headaches (20.7%) primarily affected adults and the elderly. It was also discovered that pollutants primarily affected the elderly, particularly in households where charcoal and firewood are used as a source of fuel for cooking.

## 4. Conclusion

The results presented in this research provide a first estimation of the impact of pollution on the sample households in the Ilorin South Local Government Area, Nigeria. Domestic energy sources may cause household air pollution. The study presents the incidence of a range of energy sources and cooking fuels among households and the concentrations of NO_2_, SO_2,_ and CO in the outdoor and indoor residential kitchens of urban and rural households in the Ilorin South Local Government Area, Kwara State, Northcentral Nigeria. Due to the unavailability of reliable clean energy sources, the majority of households (>80%) in Ilorin South operate solid fuels (charcoal and firewood) and LPG (20%) as the principal cooking fuels. Solid-fuel kitchens produce significantly more NO_2_ and SO_2_ than LPG kitchens, as indicated by the pollutants' greater concentrations in rural households. NO_2_ and SO_2_ levels frequently exceed the WHO air quality recommendations.

Although these air quality standards have been established for urban and rural indoor pollution, higher concentrations of these pollutants affect the lungs, which cause chronic obstructive pulmonary disease (COPD), chronic bronchitis, adverse reproductive outcomes, and pregnancy-related problems, such as stillbirth, low birth weight, and lung cancer, as reported in the literature. Finally, gaseous pollutants are more prevalent in households that use firewood and charcoal for cooking, where space confinement, noncross ventilation, and harmful activities within and around the home contribute to the increase in pollutant concentrations. It is recommended that households that use charcoal and firewood should use clean and environmentally friendly fuel (fuel with less or no emission of pollutants), while all cooking activities should always be done in a well-ventilated environment. Education of residents on modalities should be encouraged, especially in rural areas, on how to reduce exposure to pollutants.

The study has limitations, including a small sample size (for monitoring) and a fair response rate (for the survey). As a result, more research with greater population coverage will be needed in the future to determine the home level of pollutants over a longer period of time. This will help policymakers in Nigeria focus on reducing the environmental and health risks connected with the use of certain household kitchen fuels and high levels of indoor NO_2_, SO_2_, and CO. Generally, the use of LPG as a source of energy for cooking should be encouraged in developing countries to reduce the emissions of pollutants into the indoor air, which eventually will improve the health of the populace.

## Figures and Tables

**Table 1:** Interpretation of the AQI values.

Index values	AQI category	AQI rating	CO (ppm)	NO_2_ (ppm)	SO_2_ (ppm)
0–50	Good	A	0–4.4	0–0.053	0–0.035
51–100	Moderate	B	4.5–9.4	0.054–0.1	0.036–0.075
101–150	Unhealthy for sensitive groups	C	9.5–12.4	0.101–0.36	0.076–0.185
151–200	Unhealthy	D	12.5–15.4	0.361–0.64	0.186–0.304
201–300	Very unhealthy	E	15.5–30.4	0.65–1.24	0.305–0.604
301–500	Hazardous	F	30.5–50.4	1.25–2.04	0.605–1.004

**Table 2:** The selected locations, indoor sampling points, and their coordinates.

Wards	Sampling points (SP)	Coordinates
Akanbi I (fufu) SP 1	SP 1_I	8°27′6″N, 4°42′56″E
	SP 1_II	8°27′5″N, 4°42′55″E
	SP 1_III	8°27′3″N, 4°42′54″E

Akanbi II (oje) SP 2	SP 2_I	8°26′57″N, 4°44′0″E
	SP 2_II	8°27′55″N, 4°42′55″E
	SP 2_III	8°26′56″N, 4°44′2″E

Akanbi III (Gaa-akanbi) SP 3	SP 3_I	8°27′50″N, 4°34′52″E
	SP 3_II	8°27′52″N, 4°34′52″E
	SP 3_III	8°27′51″N, 4°34′50″E

Akanbi IV (tanke) SP 4	SP 4_I	8°28′51″N, 4°36′53″E
	SP 4_II	8^0^30′53″N, 4°36′54″E
	SP 4_III	8°28′51″N, 4°36′53″E

Akanbi V (sango) SP 5	SP 5_I	8^0^30′56″N, 4°35′18″E
	SP 5_II	8^0^30′32″N, 4°35′10″E
	SP 5_III	8^0^30′35″N, 4°35′16″E

Okaka I (taiwo-isale) SP 6	SP 6_I	8°28′57″N, 4°33′1″E
	SP 6_II	8°28′56″N, 4°33′3″E
	SP 6_III	8°28′54″N, 4°33′5″E

Okaka II (oke-aluko) SP 7	SP 7_I	8°29′9″N, 4°33′9″E
	SP 7_II	8°29′7″N, 4°33′7″E
	SP 7_III	8°29′6″N, 4°33′5″E

Balogun Fulani I (emirs road) SP 8	SP 8_I	8°29′16″N, 4°33′45″E
	SP 8_II	8°29′10″N, 4°33′40″E
	SP 8_III	8°29′15″N, 4°33′37″E

Balogun Fulani II (isale maliki) SP 9	SP 9_I	8°29′49″N, 4°33′36″E
	SP 9_II	8°29′47″N, 4°33′33″E
	SP 9_III	8°29′50″N, 4°33′31″E

Balogun Fulani III (opomalu) SP 10	SP 10_I	8°29′26″N, 4°33′30″E
	SP 10_II	8°29′22″N, 4°33′26″E
	SP 10_III	8°29′19″N, 4°33′30″E

Oke Ogun (edun) SP 11	SP 11_I	8°29′24″N, 4°33′13″E
	SP 11_II	8°29′23″N, 4°33′10″E
	SP 11_III	8°29′20″N, 4°33′11″E

SP 1–5 represents zone I; SP 6–7 represents zone 2; SP 8–10 represents zone 3; and SP 11 represents zone 4; SP- Sampling points.

**Table 3:** Concentrations of NO_2_ in parts per million (ppm) and their AQI rating in selected households across the sampling points.

SP	*Morning*						*Evening*					
	LPG	AQI	Charcoal	AQI	Firewood	AQI	LPG	AQI	Charcoal	AQI	Firewood	AQI
SP 1	0.029 ± 0.001	A	0.066 ±0.002	B	0.139 ±0.001	C	0.031 ±0.002	A	0.067 ±0.040	B	0.178 ±0.001	C
SP 2	0.012 ± 0.000	A	0.061 ±0.001	B	0.103 ±0.000	C	0.025 ±0.003	A	0.062 ±0.005	B	0.104 ±0.000	C
SP 3	0.029 ± 0.001	A	0.055 ±0.003	B	0.352 ±0.002	C	0.028 ±0.000	A	0.056 ±0.001	B	0.353 ±0.004	C
SP 4	0.099 ± 0.002	B	0.119 ± 0.000	C	0.178 ±0.000	C	0.101 ±0.001	C	0.122 ±0.001	C	0.178 ±0.000	C
SP 5	0.049 ± 0.000	A	0.098 ±0.003	B	0.189 ±0.002	C	0.050 ±0.001	A	0.097 ±0.003	B	0.189 ±0.000	C
SP 6	0.030 ± 0.001	A	0.096 ±0.000	B	0.475 ±0.001	D	0.034 ± 0.003	A	0.098 ±001	B	0.475 ±0.002	D
SP 7	0.022 ± 0.003	A	0.053 ±0.000	A	0.474 ±0.002	D	0.036 ± 0.001	A	0.054 ±0.003	B	0.476 ±0.003	D
SP 8	0.040 ± 0.001	A	0.073 ±0.002	B	0.104 ±0.000	C	0.042 ± 0.000	A	0.075 ±0.002	B	0.106 ±0.000	C
SP 9	0.034 ± 0.000	A	0.092 ± 0.000	B	0.441 ±0.000	D	0.035 ± 0.001	A	0.094 ±0.003	B	0.443 ±0.000	D
SP 10	0.036 ± 0.000	A	0.070 ±0.003	B	0.089 ±0.000	B	0.040 ± 0.003	A	0.071 ±0.000	B	0.090 ±0.000	B
SP 11	0.033 ± 0.003	A	0.060 ±0.000	B	0.077 ±0.000	B	0.035 ± 0.000	A	0.061 ±0.003	B	0.079 ±0.003	B

A, good, B, moderate, C, unhealthy for sensitive groups, D, unhealthy, E, very unhealthy, and F, hazardous. SP, sampling points, LPG, liquefied petroleum gas, AQI, air quality index. Each value is an average ± standard deviation concentration value of the 3 households sampled within the sampling points.

**Table 4:** Average concentration of SO_2_ in ppm and their air quality index in the selected households.

SP	*Morning*						*Evening*					
	LPG	AQI	Charcoal	AQI	Firewood	AQI	LPG	AQI	Charcoal	AQI	Firewood	AQI
SP 1	0.189 ±0.02	D	0.367 ±0.13	E	0.478 ±0.06	E	0.204 ±0.06	D	0.397 ±0.01	E	0.502 ±0.16	E
SP 2	0.150 ±0.06	C	0.364 ±0.02	E	0.478 ±0.03	E	0.166 ±0.06	C	0.413 ±0.02	E	0.491 ±0.13	E
SP 3	0.155 ±0.03	C	0.311 ±0.06	E	0.405 ±0.02	E	0.185 ±0.05	C	0.373 ± 0.05	E	0.484 ±0.11	E
SP 4	0.201 ±0.03	D	0.384 ±0.05	E	0.501 ±0.03	E	0.207 ±0.01	D	0.389 ±0.01	E	0.560 ±0.15	E
SP 5	0.252 ±0.01	D	0.276 ±0.02	D	0.593 ±0.05	E	0.272 ±0.01	D	0.491 ±0.02	E	0.647 ±0.13	F
SP 6	0.243 ±0.00	D	0.382 ±0.05	E	0.487 ±0.03	E	0.273 ±0.03	D	0.451 ±0.01	E	0.532 ±0.02	E
SP 7	0.229 ±0.02	D	0.305 ±0.03	E	0.457 ±0.02	E	0.245 ±0.02	D	0.354 ±0.04	E	0.515 ±0.03	E
SP 8	0.182 ±0.02	C	0.237 ±0.02	D	0.327 ±0.03	E	0.204 ±0.05	D	0.257 ±0.01	D	0.395 ±0.04	E
SP 9	0.247 ±0.02	D	0.352 ±0.01	E	0.441 ±0.02	E	0.272 ±0.01	D	0.384 ±0.12	E	0.467 ±0.04	E
SP 10	0.229 ±0.03	D	0.300 ±0.00	D	0.353 ±0.04	E	0.230 ±0.03	D	0.318 ±0.01	E	0.374 ±0.12	E
SP 11	0.248 ±0.00	D	0.358 ±0.02	E	0.435 ±0.03	E	0.258 ±0.01	D	0.373 ±0.02	E	0.491 ±0.03	E

A, good, B, moderate, C, unhealthy for sensitive groups, D, unhealthy, E, very unhealthy, and F, hazardous. SP, sampling points, LPG, liquefied petroleum gas, AQI, air quality index. Each value is an average ± standard deviation concentration value of the 3 households sampled within the sampling points.

**Table 5:** Average concentration of CO in ppm and their air quality index (AQI) in the selected households.

SP	*Morning*						*Evening*					
	LPG	AQI	Charcoal	AQI	Firewood	AQI	LPG	AQI	Charcoal	AQI	Firewood	AQI
SP 1	6.83 ±0.16	B	31.03 ±0.11	F	31.23 ±0.13	F	8.00 ±0.00	B	43.03 ±0.12	F	57.83 ±0.01	F
SP 2	10.40 ±0.05	C	21.20 ±0.16	E	23.58 ±0.00	E	11.20 ± 0.05	C	22.40 ±0.00	E	27.84 ±0.05	E
SP 3	4.98 ±0.13	B	9.90 ±0.05	C	12.30 ±0.11	C	5.59 ±0.16	B	10.11 ±0.05	C	13.32 ±0.01	D
SP 4	7.00 ±0.05	B	33.05 ±0.11	F	48.20 ±0.13	F	9.00 ±0.05	B	41.28 ±0.11	F	50.52 ±0.05	F
SP 5	10.00 ±0.11	C	30.20 ±0.05	E	39.20 ±0.11	F	12.00 ±0.05	C	28.20 ±0.13	E	48.20 ±0.02	F
SP 6	3.80 ±0.04	A	25.29 ±0.11	E	30.10 ±0.06	E	4.16 ±0.11	A	27.21 ±0.13	E	32.83 ±0.11	F
SP 7	3.01 ±0.00	A	35.90 ±0.05	F	42.53 ±0.13	F	3.44 ± 0.11	A	39.01 ±0.00	F	43.90 ± 0.04	F
SP 8	4.17 ±0.11	A	20.75 ±0.11	E	25.74 ±0.00	E	4.27 ±0.11	A	23.21 ±0.05	E	37.50 ±0.05	F
SP 9	4.36 ±0.11	A	30.58 ±0.05	F	33.12 ±0.05	F	5.00 ±0.00	B	36.45 ±0.08	F	39.40 ± 0.05	F
SP 10	5.69 ±0.05	B	20.50 ±0.00	E	23.69 ±0.11	E	8.33 ±0.00	B	21.87 ±0.11	E	27.34 ±0.04	E
SP 11	8.43 ±0.03	B	29.50 ±0.05	E	32.33 ±0.11	F	10.01 ± 0.05	C	31.90 ±0.05	F	36.97 ±0.11	F

A, good, B, moderate, C, unhealthy for sensitive groups, D, unhealthy, E, very unhealthy, and F, hazardous. SP, sampling points, LPG, liquefied petroleum gas, AQI, air quality index. Each value is an average ± standard deviation concentration value of the 3 households sampled within the sampling points.

**Table 6:** The estimate of the model parameters for NO_2_.

*Model*	*Unstandardized Coefficients*	*Standardized Coefficients*				*95% Confidence Interval for B*	
	*B*	*Std. Error*	*Beta*	*T*	*Sig.*	Lower Bound	Upper Bound
(Constant)	0.034	0.025		1.37	0.188	-0.018	0.087
z2	0.003	0.027	0.139	1.08	0.290	-0.028	0.086
z3	0.020	0.027	0.097	0.759	0.458	-0.036	0.077
z4	-0.049	0.027	-0.235	-1.84	0.084	-0.106	0.007
EVNG	0.007	0.019	0.039	0.37	0.710	-0.033	0.047
CHAR	0.039	0.023	0.200	1.66	0.115	-0.010	0.087
FIWD	0.177	0.023	0.919	7.612	0.000	0.128	0.226

a. dependent variable: concentration of NO_2._ EVNG, evening; CHAR, charcoal; FIWD, firewood.

**Table 7:** The estimate of the model parameters for SO_2_.

	Unstandardized Coefficients	Standardized Coefficients				95% Confidence Interval for B	
Model	B	Std. Error	Beta	T	Sig.	Lower Bound	Upper Bound
(Constant)	0.06	0.004		14.8	0.000	0.052	0.069
z2	−0.006	0.004	−0.10	−1.41	0.18	−0.015	0.003
z3	−0.008	0.004	−0.14	−1.82	0.086	−0.017	0.001
z4	0.000	0.004	−0.003	−0.038	0.97	−0.009	0.009
EVNG	0.009	0.003	0.17	2.79	0.012	0.002	0.015
CHAR	0.032	0.004	0.59	8.52	0.000	0.024	0.04
FIWD	0.006	0.004	1.008	15.6	0.000	0.051	0.07

a. dependent variable: SO_2._ EVNG, evening; CHAR, charcoal; FIWD, firewood.

**Table 8:** The estimate of the model parameters for CO.

	Unstandardized Coefficients	Standardized Coefficients				95% Confidence Interval for B	
Model	B	Std. Error	Beta	T	Sig.	Lower Bound	Upper Bound
(Constant)	6.7	1.7		3.9	0.001	3.1	10.4
z2	1.1	1.9	0.0	0.6	0.550	−2.8	5.0
z3	3.3	1.9	0.1	1.8	0.090	−0.6	7.2
z4	−1.4	1.9	−0.0	−0.8	0.450	−5.3	2.5
EVNG	4.7	1.3	0.2	3.5	0.002	1.8	7.4
CHAR	21.7	1.6	0.8	13.5	0.000	18.3	25.0
FIWD	30.4	1.6	1.1	18.9	0.000	26.9	33.7

a. dependent variable: CO. EVNG, evening; CHAR, charcoal; FIWD, firewood.

**Table 9:** Health conditions discovered in some of the respondents.

Health Problem	Frequency	Percentage (%)	Age Category(year)

Cough	15	17.2	47–60
Dizziness	8	9.2	50–55
Irritation of eye	10	11.5	40–45
Cold	3	3.4	30–55
Headache	18	20.7	25–55
Fatigue	7	8.0	35–50
Sneezing	10	11.5	40–55
Chest pain	5	5.7	45–58
Poor breathing	3	3.4	45–60
Fever	3	3.4	30–50
Malaria	5	5.7	25–55
Total	87	100	

## Data Availability

1. The (Air Quality Index and Health Risk Assessment) data used to support the findings of this study are included within the article 2. The (Raw data for the concentrations of the pollutants) data used to support the findings of this study are available from the corresponding author upon request.

## Supplementary Materials

Supplementary Materials Supplementary 1 (S1): Air quality index values. Supplementary 2 (S2): Mean inhalation rate and mean body weight for the exposed groups. Supplementary 3 (S3): Result of CO using MSA Altair 5x Multi Gas sensor. Supplementary 4 (S4): Result of NO_2_ using a UV-visible spectrophotometer. Supplementary 5 (S5): Result of SO_2_ using a conductivity meter. Supplementary 6 (S6): Questionnaire sample.
