# Supplementary material for: Assessment of Kitchen Air Pollution: Health Implications for the Residents of Ilorin South, Nigeria
Source: J Environ Public Health. 2022 Aug 17;2022:7689141. doi: 10.1155/2022/7689141 (PMC9402372; doi:10.1155/2022/7689141)
Supplement: Supplementary Materials — Supplementary 1 (S1): Air quality index values. Supplementary 2 (S2): Mean inhalation rate and mean body weight for the exposed groups. Supplementary 3 (S3): Result of CO using MSA Altair 5x Multi Gas sensor. Supplementary 4 (S4): Result of NO2 using a UV-visible spectrophotometer. Supplementary 5 (S5): Result of SO2 using a conductivity meter. Supplementary 6 (S6): Questionnaire sample. [file 7689141.f1.doc]

***Supplementary 1 (S1): Air Quality Index Values***

| **Air Quality (AQI) Values** | **Levels of Health Concern** | **Color** |
| --- | --- | --- |
| **when the AQI is on this range** | **Air Quality Conditions are:** | **As symbolized by this color** |
| 0 to 50 | Good | **Green** |
| 51 to 100 | Moderate | **Yellow** |
| 101 to 150 | Unhealthy for sensitive groups | **Orange** |
| 151 to 200 | Unhealthy | **Red** |
| 201 to 300 | Very unhealthy | **Purple** |
| 301 to 500 | Hazardous | **Maroon** |

S1 is a table of AQI values, their respective level of health concern symbolized with colours that was used in this research.

**Supplementary 2 (S2): Mean Inhalation Rate and Mean Body Weight for the Exposed**

**Groups**

| Exposed group | (Acute exposure) Mean inhalation rate (m^3^/hour) | Mean body weight (kg) |
| --- | --- | --- |
| Infant (birth to 1 year)  Child (6-12 years)  Adult (19-75 years) | 0.3  1.2  1.2 | 11.3  45.3  71.8 |

Source: Adapted from (Matooane and Diab*,* 2003; USEPA, 1997)

S2 is the supplementary table that showed the mean inhalation rate and mean body weight for exposed groups in this research similar to what was seen from reviewed literature.

**Supplementary 3 (S3): Result of CO Using MSA Altair 5x Multi Gas Sensor**

| **Sampling points** | **Morning** | | | **Evening** | | |
| --- | --- | --- | --- | --- | --- | --- |
|  | **Gas** | **Charcoal** | **Firewood** | **Gas** | **Charcoal** | **Firewood** |
| Akanbi I  Fufu | 6.83 | 31.03 | 31.23 | 8.00 | 43.03 | 57.83 |
| Akanbi II  Oje | 10.40 | 21.20 | 23.58 | 11.20 | 22.40 | 27.84 |
| Akanbi III  Gaa-Akanbi | 4.98 | 9.90 | 12.30 | 5.59 | 10.11 | 13.32 |
| Akanbi IV  Tanke | 7.00 | 33.05 | 48.20 | 9.00 | 41.28 | 50.52 |
| Akanbi V  Sango | 10.00 | 30.20 | 39.20 | 12 | 28.20 | 48.20 |
| Balogun Fulani I  Emirs Road | 3.80 | 25.29 | 30.10 | 4.16 | 27.21 | 32.83 |
| Balogun Fulani II  Isale Maliki | 3.01 | 35.90 | 42.53 | 3.44 | 39.01 | 43.90 |
| Balogun Fulani III  Opomalu | 4.17 | 20.75 | 25.74 | 4.27 | 23.21 | 37.50 |
| Okaka I  Taiwo Isale | 4.36 | 30.58 | 33.12 | 5.00 | 36.45 | 39.40 |
| Okaka II  Oke-Aluko | 5.69 | 20.50 | 23.69 | 8.33 | 21.87 | 27.34 |
| Oke-Ogun  Edun | 8.43 | 29.50 | 32.33 | 10.01 | 31.90 | 36.97 |

The result of CO gas assessed from different sources of cooking using MSA Altair 5X Multi Gas Sensor of various sampling points (both morning and evening) is shown in Table S3.

**Supplementary 4 (S4): Result of NO_2_ Using UV-Visible Spectrophotometer**

| **Sampling points** | **Morning** | | | **Evening** | | |
| --- | --- | --- | --- | --- | --- | --- |
|  | **Gas** | **Charcoal** | **Firewood** | **Gas** | **Charcoal** | **Firewood** |
| Akanbi I Fufu | 0.082 | 0.182 | 0.382 | 0.086 | 0.185 | 0.489 |
| Akanbi II Oje | 0.032 | 0.168 | 0.283 | 0.069 | 0.171 | 0.284 |
| Akanbi III Gaa-Akanbi | 0.079 | 0.151 | 0.966 | 0.078 | 0.153 | 0.969 |
| Akanbi IV Tanke | 0.273 | 0.328 | 0.488 | 0.276 | 0.336 | 0.489 |
| Akanbi V Sango | 0.136 | 0.268 | 0.521 | 0.138 | 0.267 | 0.520 |
| Balogun Fulani I  Emirs Road | 0.083 | 0.262 | 1.304 | 0.094 | 0.269 | 1.308 |
| Balogun Fulani II  Isale Maliki | 0.061 | 0.146 | 1.299 | 0.100 | 0.149 | 1.305 |
| BAlogun Fulani III  Opomalu | 0.111 | 0.201 | 0.284 | 0.114 | 0.205 | 0.291 |
| Okaka I Taiwo Isale | 0.092 | 0.251 | 1.211 | 0.096 | 0.257 | 1.215 |
| Okaka II Oke-Aluko | 0.100 | 0.192 | 0.243 | 0.109 | 0.196 | 0.246 |
| Oke-Ogun Edun | 0.090 | 0.163 | 0.212 | 0.097 | 0.168 | 0.216 |

S4 showed the results of NO_2_ gas assessed from different sources of cooking using UV-Visible Spectrophotometer measured absorbance @ 550 nm of samples from various sampling points (both morning and evening).

**Supplementary 5 (S5): Result of SO_2_ Using Conductivity Metre**

| **Sampling points** | **Morning** | | | **Evening** | | |
| --- | --- | --- | --- | --- | --- | --- |
|  | **Gas** | **Charcoal** | **Firewood** | **Gas** | **Charcoal** | **Firewood** |
| Akanbi I Fufu | 3.90 | 7.58 | 9.86 | 4.20 | 8.20 | 10.36 |
| Akanbi II Oje | 3.10 | 7.50 | 9.86 | 3.42 | 8.52 | 10.12 |
| Akanbi III Gaa-Akanbi | 3.20 | 6.41 | 8.35 | 3.82 | 7.69 | 9.98 |
| Akanbi IV Tanke | 4.15 | 7.92 | 10.52 | 4.28 | 8.02 | 11.56 |
| Akanbi V Sango | 5.20 | 9.82 | 12.24 | 5.62 | 10.12 | 13.34 |
| Balogun Fulani I  Emirs Road | 5.01 | 7.89 | 10.05 | 5.63 | 9.30 | 10.97 |
| Balogun Fulani II  Isale Maliki | 4.73 | 6.30 | 9.43 | 5.06 | 7.30 | 10.63 |
| BAlogun Fulani III  Opomalu | 3.76 | 4.89 | 6.75 | 4.21 | 5.30 | 8.15 |
| Okaka I Taiwo Isale | 5.09 | 7.26 | 9.10 | 5.61 | 7.92 | 9.63 |
| Okaka II Oke-Aluko | 4.72 | 6.19 | 7.28 | 4.75 | 6.57 | 7.72 |
| Oke-Ogun Edun | 5.12 | 7.39 | 8.97 | 5.33 | 7.69 | 10.12 |

The result of SO_2_ gas assessed from different sources of cooking using conductivity metre measured in micro Siemens per cm of various sampling points both morning and evening is shown in S5.

**Supplementary 6 (S6): Questionnaire Sample**

A questionnaire was developed for the assessment of information from the residents to elicit information on the health impacts of the pollutants to the residents.

**Directions:**

Please tick within the box your response and where possible, write your response.

*Thank you for your co-operation.*

**SECTION A: RESPONDENT’S DEMOGRAPHIC INFORMATION**

Gender: Female ( ) Male ( )

Address:

Age: 20 – 30 ( ) 31 – 40 ( ) 41 – 50 ( ) 51 and above ( )

Income Level: N18,000 – N 30,000 ( ), N 31,000 – N 50,000 ( ), N 51, 0000 and Above ( )

Level of Education: None Formal Education ( ), Primary ( ), SSCE ( ), graduate ( ) above ( )

Type of Kitchen: Attached ( ) detached ( )

Time of cooking: 30 minutes ( ) 1 hour ( ) 2 hours ( ) Above ( )

Number of people in the household: 1-3 ( ) 4-6 ( ) Above ( )

SECTION B

SIGN OF THE FOLLOWING EXPERIENCED BY THE RESIDENTS

| Symptoms | Yes/No |
| --- | --- |
| Cough |  |
| Sneezing |  |
| Irritation of the eyes |  |
| Cold |  |
| Cold |  |
| fatique |  |
| Chest pain |  |
| Poor breathing |  |
| fever |  |
| malaria |  |
| Did any of the resident been admitted to the hospital? |  |
| Do you use generator |  |
| Do you open the windows while cooking |  |
| Do you open the doors while cooking |  |
| Do you smoke |  |
| Does anyone smoke at your surroundings |  |
